# Biogenic selenium nanoparticles: characterization, antimicrobial activity and effects on human dendritic cells and fibroblasts

**DOI:** 10.1111/1751-7915.12374

**Published:** 2016-06-20

**Authors:** Eleonora Cremonini, Emanuele Zonaro, Marta Donini, Silvia Lampis, Marzia Boaretti, Stefano Dusi, Paola Melotti, Maria M. Lleo, Giovanni Vallini

**Affiliations:** ^1^ Department of Diagnostic and Public HealthUniversity of VeronaStrada Le Grazie 837134VeronaItaly; ^2^ Department of BiotechnologyUniversity of VeronaStrada Le Grazie 1537134VeronaItaly; ^3^ Department of MedicineUniversity of VeronaStrada Le Grazie 837134VeronaItaly; ^4^ Cystic Fibrosis Regional CenterAOUI VeronaVeronaItaly

## Abstract

Tailored nanoparticles offer a novel approach to fight antibiotic‐resistant microorganisms. We analysed biogenic selenium nanoparticles (SeNPs) of bacterial origin to determine their antimicrobial activity against selected pathogens in their planktonic and biofilm states. SeNPs synthesized by Gram‐negative *Stenotrophomonas maltophilia* [Sm‐SeNPs(−)] and Gram‐positive *Bacillus mycoides* [Bm‐SeNPs(+)] were active at low minimum inhibitory concentrations against a number of clinical isolates of *Pseudomonas aeruginosa* but did not inhibit clinical isolates of the yeast species *Candida albicans* and *C. parapsilosis*. However, the SeNPs were able to inhibit biofilm formation and also to disaggregate the mature glycocalyx in both *P. aeruginosa* and *Candida* spp. The Sm‐SeNPs(−) and Bm‐SeNPs(+) both achieved much stronger antimicrobial effects than synthetic selenium nanoparticles (Ch‐SeNPs). Dendritic cells and fibroblasts exposed to Sm‐SeNPs(−), Bm‐SeNPs(+) and Ch‐SeNPs did not show any loss of cell viability, any increase in the release of reactive oxygen species or any significant increase in the secretion of pro‐inflammatory and immunostimulatory cytokines. Biogenic SeNPs therefore appear to be reliable candidates for safe medical applications, alone or in association with traditional antibiotics, to inhibit the growth of clinical isolates of *P. aeruginosa* or to facilitate the penetration of *P. aeruginosa* and *Candida* spp. biofilms by antimicrobial agents.

## Introduction

Resistance to antimicrobial drugs has become more widespread over the last decades resulting in a significant threat to public health. Infections caused by antibiotic‐resistant bacteria need higher doses of drugs, additional toxic treatments and extended hospital stays, and ultimately result in increased mortality (Gadakh and Van Aerschot, 2015). Despite the need for new antibiotics, only limited resources have been allocated by the pharmaceutical industry to support the discovery of new antibacterial agents, largely because the financial returns are likely to be small. To prevent or overcome antimicrobial resistance, non‐antibiotic therapies will be necessary to treat bacterial infections and alternative strategies that show promise for the management of resistant infections are already under investigation (Beyth *et al*., 2015; Gill *et al*., 2015).

Most antibiotics that are active against free microbes are less effective against the same species when present as a biofilm. This is a particular concern because microbial biofilms play a pivotal role in many infections, and biofilm‐related traits may confer high‐level antibiotic resistance in microbial communities (Penesyan *et al*., 2015). The biofilm matrix can act as a mechanical barrier, hindering the penetration of antibacterial agents and immune response effectors. However, bacteria can also become highly resistant to antibiotics as a result of nutrient limitation or the emergence of a persistent but non‐growing phenotype that allows microbial cells to cope efficiently with environmental stresses, including antibiotic challenge (Grant and Hung, 2013).

Strategies that prevent biofilm formation or dispersal are not fully effective in the absence of a treatment that also counters the growth of individual cells. For this reason, a combination of anti‐biofilm therapy along with traditional antibiotics that target bacterial growth offers a promising approach for the control of biofilm‐related infectious diseases. In such combination approaches, the anti‐biofilm agent would force microbes into their planktonic growth state, thus facilitating the targeting of pathogens at the cellular level with traditional antibiotics (Kostakioti *et al*., 2013).

Recent developments in nanotechnology allow the production of tailored metal/metalloid nanoparticles with physicochemical properties that can inhibit microorganisms. These nanoparticles have been shown to overcome existing drug resistance mechanisms, including slow drug uptake and accelerated efflux, biofilm formation and intracellular bacterial parasitism (Pelgrift and Friedman, 2013). In this context, selenium nanoparticles (SeNPs) possess antibacterial, antiviral and antioxidant properties, suggesting they could be suitable as therapeutic candidates to combat infectious diseases. In particular, nanostructured particles can be synthesized using bacterial and fungal cells as biological catalysts, providing a non‐toxic and environmentally beneficial approach for the production of nanoparticles, including SeNPs (Xiangqian *et al*., 2011). Several microbial strains can reduce the toxic selenite oxyanion to the less toxic elemental selenium through the formation of either intracellular or extracellular SeNPs, with a typical spherical shape and a diameter of 50–400 nm (Lampis *et al*., 2014). Furthermore, recent studies with biogenic SeNPs have demonstrated that the particles have anti‐biofilm activity against clinical isolates of bacterial pathogens (Shakibaie *et al*., 2015). However, their antibacterial effects are not fully understood and their potential toxicity towards human tissues requires further investigation.

Another potential limitation affecting the clinical application of metal/metalloid nanoparticles is the ability of some nanostructured materials to stimulate the release, by dendritic cells (DCs) and other cells in the immune system, of reactive oxygen species and/or chemical mediators that trigger unwanted side‐effects such as hypersensitivity reactions, autoimmune diseases and inflammatory responses (Chang and Gershwin, 2010; Di Gioacchino *et al*., 2011). In particular, activated DCs produce oxygen free radicals that can cause severe tissue damage (Vulcano *et al*., 2004; Donini *et al*., 2007), and may also release cytokines that play a key role in the induction of inflammatory and immune responses (Elmquist *et al*., 1997; Suffredini *et al*., 1999; Granucci *et al*., 2008; Schäkel, 2009; Vignali and Kuchroo, 2012). Therefore, nanoparticle candidates suitable for clinical applications must not induce DC activation or have toxic effects against cells of the immune system and other tissues.

Here, SeNPs generated by two environmental bacterial isolates, namely a strain of the Gram‐positive species *Bacillus mycoides* (Bm‐SeNPs(+)) and a strain of the Gram‐negative species *Stenotrophomonas maltophilia* (Sm‐SeNPs(−)), were compared with chemically synthesized SeNPs (Ch‐SeNPs) in terms of their physical and biological properties, including toxicity and immunostimulatory activity *in vitro* against cultures of human DCs and fibroblasts. The antibacterial and anti‐biofilm characteristics of the SeNPs were tested against clinical strains of *Pseudomonas aeruginosa* and clinical isolates of two *Candida* species.

## Results and discussion

### Biosynthesis and characterization of SeNPs

**Figure 1:**
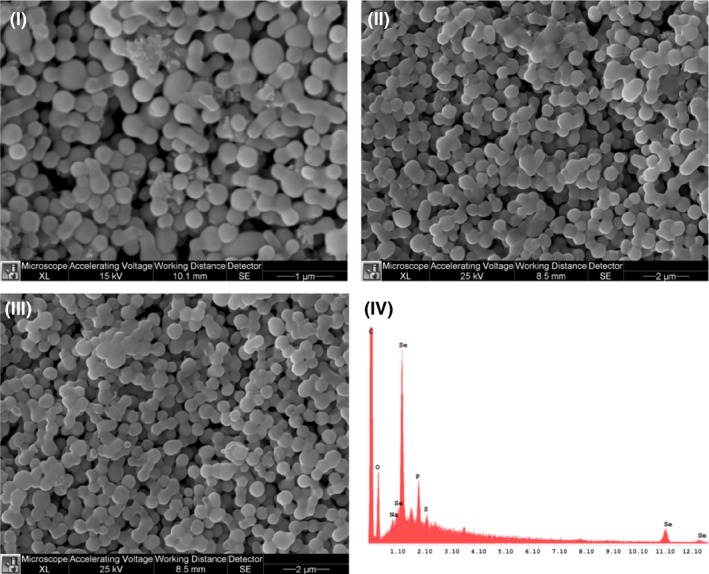
Scanning electron microscopy analysis of SeNPs produced by *Stenotrophomonas maltophilia* SeITE02 (I), SeNPs produced by *Bacillus mycoides* SeITE01 (II) and chemically synthesized SeNPs (III). EDX analysis of biogenic SeNPs (IV).

Biogenic SeNPs were produced by exploiting the selenite reduction capability of two different environmental bacterial isolates, namely *B. mycoides* SeITE01 and *S. maltophilia* SeITE02. The biogenic SeNPs were compared with synthetic Ch‐SeNPs in terms of their physicochemical characteristics. Scanning electron microscopy (SEM) analysis indicated that all three SeNPs were spherical and EDAX microanalysis of the purified SeNPs revealed the characteristic selenium absorption peaks at 1.37 (SeLα), 11.22 (SeKα) and 12.49 keV (SeKβ) (Fig. 1). Moreover, SeNPs differently synthesized showed different elemental composition (Table 1). For instance, biogenic SeNPs showed a selenium percentage in weight of 11.01% and 9.26% for Sm‐SeNPs(−) and Bm‐SeNPs(+) respectively. On the other hand, Ch‐SeNPs exhibited a higher percentage in selenium, 31.61%. Furthermore, the composition of biogenic SeNPs, which were rich in C, O, P and S, suggests the presence of biological macromolecules surrounding the nanomaterials. Based on the presence and percentage of C, O and S, it is possible to hypothesize that biogenic SeNPs cap include proteins or enzymes and also some cellular residues and membrane phospholipids (P peaks). In particular, a tentative composition of the biomolecular capping surrounding SeNPs biosynthesized by *S. maltophilia* SeITE02 has been already reported. Actually, Fourier transform infrared spectroscopy (FTIR) analysis of Sm‐SeNPs(−) evidenced the presence of proteins, lipids and carbohydrates associated with the nanomaterial (Lampis *et al*., 2016).

**Table 1:** Elemental composition of Ch‐SeNPs, Sm‐SeNPs(−) and Bm‐SeNPs(+) calculated through EDX analysis

Element	Ch‐SeNPs	Sm‐SeNPs(−)	Bm‐SeNPs(+)
C	60.91	73.13	75.75
O	4.97	10.44	10.82
Se	31.61	11.01	9.26
P	1.88	4.42	3.14
S	0.63	1.00	1.04

In Ch‐SeNPs, the same elements are present in different percentage: 60.91% in weight of C, 4.97% in weight of O, 1.88% in weight of P and 0.63% in weight of S: this is probably due to the procedure used for the synthesis.

**Figure 2:**
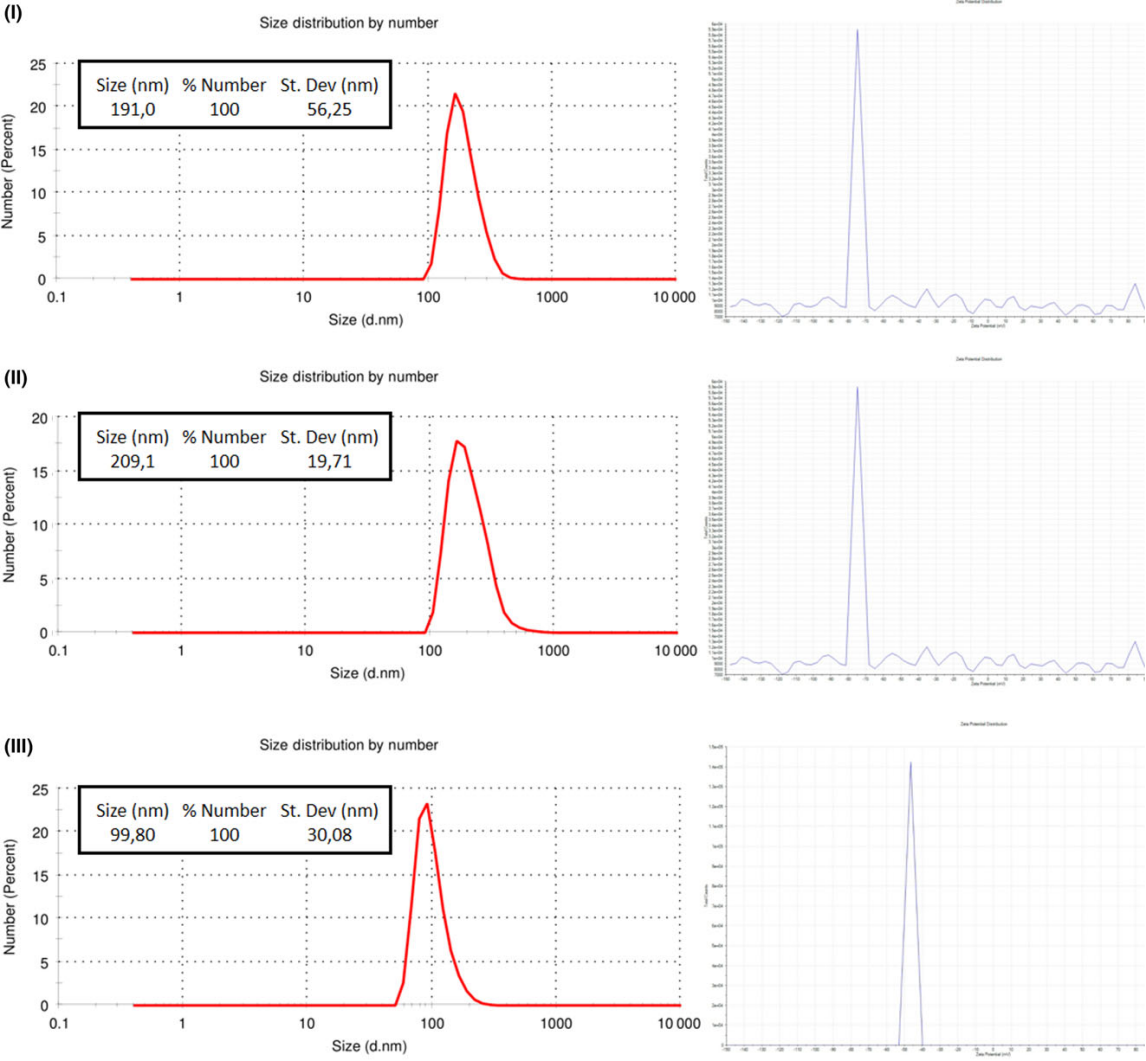
Dynamic light scattering analysis and zeta potential of SeNPs produced by *Stenotrophomonas maltophilia* SeITE02 (I), SeNPs produced by *Bacillus mycoides* SeITE01 (II) and chemically synthesized SeNPs (III).

Dynamic light scattering measurements indicated average sizes of 170.6 ± 35.12 nm for the Sm‐SeNPs(−), 160.6 ± 52.24 nm for the Bm‐SeNPs(+) and 102.5 ± 29.44 nm for the Ch‐SeNPs (Fig. 2).

All three SeNPs generated large negative zeta potentials (between −70 and −80 mV) in solution (Fig. 2) suggesting they are unlikely to form aggregates as a consequence of their electrostatic stability. Neutral and negatively charged NPs tend to have long half‐lives in human serum and are not taken up by cells in a non‐specific manner (Alexis *et al*., 2008). This is important in the context of potential *in vivo* applications as antimicrobial reagents.

### Determination of the minimum inhibitory concentration for SeNPs against *Pseudomonas aeruginosa* PAO1

In order to evaluate the effect of biogenic and synthetic SeNPs as antimicrobial agents, as well as to study the putative role of the biomolecular cap of the biogenic nanoparticles, the determination of minimum inhibitory concentration (MIC) values was first carried out against the reference strain *Pseudomonas aeruginosa* PAO1 (Table 2). MIC determination was carried out for the biogenic SeNPs, Sm‐SeNPs(−) and Bm‐SeNPs(+), the chemically synthesized Ch‐SeNPs, the Ch‐SeNPs exposed to cell free extract (CFX) of *S. maltophilia* SeITE02 (CFX(Sm)‐SeNPs) and *B. mycoides* SeITE01 (CFX(Bm)‐SeNPs)) and CFX of *S. maltophilia* SeITE02 (CFX(Sm)) and *B. mycoides* SeITE01 (CFX(Bm)) alone.

**Table 2:** Minimum inhibitory concentration (MIC) of Ch‐SeNPs, Ch2‐SeNPs, Sm‐SeNPs(−), Bm‐SeNPs(+), CFX(Sm)‐SeNPs, CFX(Bm)‐SeNPs, CFX(Sm) and CFX(Bm) against *Pseudomonas aeruginosa* PAO1

Strain	Ch‐SeNPs MIC (μg ml^−1^)	Ch2‐SeNPs MIC (μg ml^−1^)	Sm‐SeNPs(−) MIC (μg ml^−1^)	Bm‐SeNPs(+) MIC (μg ml^−1^)	CFX(Sm)‐SeNPs MIC (μg ml^−1^)	CFX(Bm)‐SeNPs MIC (μg ml^−1^)	CFX(Sm) MIC (μl ml^−1^)	CFX(Bm) MIC (μl ml^−1^)
*Pseudomonas aeruginosa* PAO1	128	128	128	128	256	256	>512	>512

As we can see from Table 2, Ch‐SeNPs, Sm‐SeNPs(−) and Bm‐SeNPs(+) evidenced a similar MIC value of 128 μg ml^−1^. On the other hand, CFX(Sm)‐SeNPs and CFX(Bm)‐SeNPs showed a lower activity towards the reference strain, with a MIC value of 256 μg ml^−1^. Finally, CFX from both *S. maltophilia* SeITE02 and *B. mycoides* SeITE01 did not exhibit antimicrobial activity at any of the concentration tested in the present analysis. These results clearly indicate that the antimicrobial activity observed is exactly due to the nanoparticles and the biomolecular cap to them associated through the biosynthetic mechanism. Several studies have already analysed the antimicrobial activity of SeNPs synthesized chemically towards different pathogenic bacterial strains. For instance, chemically synthesized SeNPs were able to inhibit the growth of *Staphylococcus aureus* (Tran and Webster, 2011), with higher efficiency than silver phosphate nanoparticles (Chudobova *et al*., 2014).

### Antimicrobial activity of SeNPs against clinical isolates of *P. aeruginosa* and *Candida* spp.

To test the antibacterial and anti‐biofilm activity of the SeNPs, we selected a series of clinical strains of *P. aeruginosa*, whose surrounding polysaccharide biofilm matrix confers resistance to eradication by antibiotics and clearance by the immune system. Such strains are recurrent in the chronic lung infections that characterize cystic fibrosis, chronic obstructive pulmonary disease and asthma (Ciofu *et al*., 2015). The multidrug‐resistant isolate *P. aeruginosa* INT was chosen to provide a particularly challenging target, whereas *P. aeruginosa* PA01 and ATCC 27853 were included as reference strains. The ability of SeNPs to inhibit bacterial growth was tested by challenging the bacterial isolates and reference strains with different concentrations of SeNPs, then determining the MIC using the agar well diffusion assay and the broth dilution method.

As shown in Table 3, the MIC of Sm‐SeNPs(−) varied widely among the different *P. aeruginosa* strains, ranging from 8 to 16 μg ml^−1^ in strains isolated from low respiratory tract infections (CFC20, CFC21, CFCA and CFCB) to 128–512 μg ml^−1^ against *P. aeruginosa* INT and both the reference strains. The MIC of Bm‐SeNPs(+) also varied among the strains but was generally 2–4 times higher than Sm‐SeNPs (Table 3). The MIC of Ch‐SeNPs indicated that these SeNPs have no effect on the growth of *P. aeruginosa* (Table 3).

**Table 3:** Minimal inhibitory concentrations (MICs) for *Stenotrophomonas maltophilia* (Sm)‐SeNPs, *Bacillus mycoides* (Bm)‐SeNPs and chemically synthesized (Ch)‐SeNPs tested against different microbial strains isolated from clinical samples. MIC values in the clinical usage range are in bold

Bacterial strain/*Candida* species	MIC (μg ml^−1^)		
	Sm‐SeNPs	Bm‐SeNPs	Ch‐SeNPs
*Pseudomonas aeruginosa* PAO1	128	128	128
*P. aeruginosa* ATCC27853	512	512	>512
*P. aeruginosa* INT	256	512	>512
*P. aeruginosa* CFC20	**16**	**64**	128
*P. aeruginosa* CFC21	**8**	**32**	128
*P. aeruginosa* CFCB	**16**	**32**	>128
*P. aeruginosa* CFCA	**16**	**64**	>128
*Candida albicans*	256	512	>512
*C. parapsilosis*	512	512	>512

The susceptibility of the reference strains *P. aeruginosa* PAO1 and ATCC 27853 to Sm‐SeNPs(−) was determined in a previous study and the reported MIC values were slightly different to those we observed (250 μg ml^−1^) (Zonaro *et al*., 2015). The difference probably reflects the distinct methods used to determine the MIC in each study and the use of different growth media, which is known to affect the toxicity of NPs (Loza *et al*., 2014).

We found that *P. aeruginosa* clinical strains CFC20, CFC21, CFCA and CFCB were susceptible to Sm‐SeNPs with MIC values in the range 2.5–20 μg ml^−1^, which is broadly in agreement with previous reports dealing with other nanoparticles (Habash *et al*., 2014; Tomita *et al*., 2014). Because these MIC values fall within the range of clinical exposures adopted during typical antibiotic treatments, biogenic SeNPs could be used to treat antibiotic‐resistant clinical strains, eventually overcoming the potential risks of antibiotic resistance (Table 3). Our analysis of antibiotic susceptibility carried out according to Clinical and Laboratory Standards Institute standard methods (Jorgensen and Ferraro, 2009) demonstrated that the four *P. aeruginosa* clinical strains were resistant to beta‐lactams (MIC values varying between 16 and ≥ 64 μg ml^−1^) and other antibiotics such as gentamicin (MIC values 8–16 μg ml^−1^), ciprofloxacin (MICs between 2 and ≥ 4 μg ml^−1^) and sulphonamides (MIC values 40–80 μg ml^−1^).

Conversely, given the much higher MIC for *P. aeruginosa* INT (> 250 μg ml^−1^) compared with the clinical strains, it is unlikely that SeNPs could solve the problem of antibiotic resistance in this isolate.

We also evaluated the anti‐fungal activity of SeNPs by testing *in vitro* their ability to inhibit the growth of *C. albicans* and *C. parapsilosis* clinical strains. The MIC values were very high (> 256 μg ml^−1^) indicating that SeNPs do not inhibit the growth of these yeast strains.

### Inhibition of *P. aeruginosa* and *Candida* spp. biofilm formation by SeNPs

To analyse the effect of SeNPs on biofilm synthesis, the *P. aeruginosa* strains and the two *Candida* species were treated for 48 h at 37°C with different concentrations of Bm‐SeNPs(+), Sm‐SeNPs(−) and Ch‐SeNPs, and biofilm formation was quantified by methylene blue staining. The percentage of biofilm inhibition was calculated by comparing the microbial cultures exposed to the SeNPs with the same strains growing in the absence of SeNPs.

The quantity of biofilm produced by *P. aeruginosa* strains CFCA and CFCB was greater than that produced by the other *P. aeruginosa* strains and by the yeast isolates, when values were averaged over six experiments (Table 4). The lowest concentrations of biogenic SeNPs (50 and 100 μg ml^−1^) inhibited biofilm synthesis by *P. aeruginosa* strains CFC20, CFC21 and CFCA by 70–90%, indicating that they are particularly sensitive to the SeNPs. In contrast, clinical strains CFCB and INT, as well as the reference strains, were more resistant to the SeNPs, showing the significant inhibition of biofilm synthesis (at least 70%) only in the presence of SeNP concentrations ≥ 250 μg ml^−1^. Table 4 also shows that Sm‐SeNPs(−) were usually more potent than Bm‐SeNPs(+), and that the synthetic Ch‐SeNPs were only active at concentrations of 250–500 μg ml^−1^ against most strains, the exception being *P. aeruginosa* CFC20, which was the most susceptible isolate tested.

**Table 4:** Percentage of biofilm synthesis inhibition in different bacteria strains caused by Sm‐SeNPs, Bm‐SeNPs and Ch‐SeNPs

Bacterial strain^a^	*Stenotrophomonas maltophilia*‐SeNPs (μg ml^−1^)				*Bacillus mycoides*‐SeNPs (μg ml^−1^)				Chemically synthesized SeNPs (μg ml^−1^)			
	50	100	250	500	50	100	250	500	50	100	250	500
*Pseudomonas aeruginosa* PAO1 (1.10)	40 ± 2.5	45 ± 3	70 ± 2.5	**96 ± 1**	33 ± 3	47 ± 6	63 ± 4.5	**95 ± 1.2**	9 ± 0.7	21 ± 2.1	74 ± 0.7	**96 ± 1.4**
*P. aeruginosa ATCC27853* (1.00)	15 ± 0.7	30 ± 0.7	41 ± 0.7	66 ± 2.1	15 ± 4.2	17 ± 2	44 ± 2.8	64 ± 1	4 ± 1.4	10 ± 0.7	35 ± 2.1	44 ± 2
*P. aeruginosa* INT (1.00)	23 ± 1	34 ± 1	59 ± 3.5	**95 ± 1**	25 ± 4.5	29 ± 3.5	49 ± 2.5	**94 ± 1.5**	2 ± 3.5	2 ± 1.4	20 ± 0.7	30 ± 0.7
*P. aeruginosa* CFC21 (1.20)	66 ± 5	**86 ± 0.5**	**95 ± 2**	**96 ± 1.5**	37 ± 5.5	66 ± 3.5	**93 ± 1.7**	**95 ± 2**	10 ± 3.5	33 ± 3.5	71 ± 1.4	65 ± 1.4
*P. aeruginosa* CFC20 (1.10)	75 ± 0.5	**82 ± 1**	**86 ± 1**	**93 ± 1.5**	72 ± 2	76 ± 0.5	**85 ± 1**	**91 ± 2**	53 ± 0.7	**81 ± 0.7**	**95 ± 1**	**95 ± 1**
*P. aeruginosa* CFCB (2.75)	31 ± 1	69 ± 3	79 ± 2	**88 ± 4**	28 ± 1.5	34 ± 5	**81 ± 4.5**	**85 ± 5**	1 ± 0.5	1 ± 0.5	77 ± 0.7	**98 ± 1**
*P. aeruginosa* CFCA (3.00)	39 ± 1	**94 ± 1**	**94 ± 3.5**	**96 ± 1**	25 ± 5	25 ± 1.5	**94 ± 0.5**	**96 ± 1**	5 ± 0.7	6 ± 0.7	**96 ± 1**	**97 ± 1.5**

a Quantity of biofilm in arbitrary units.

The percentage of inhibition was calculated relative to the quantity of biofilm formed by each strain in the absence of nanoparticles. Data are the average of results obtained in three different experiments. Percentages of inhibition higher than 80% are shown in bold.

Interestingly, the lowest SeNP dose we tested (50 μg ml^−1^) was sufficient for 60–70% biofilm inhibition in the two yeast isolates, and no significant improvement was achieved at the higher doses of 100 and 250 μg ml^−1^ (Table 5). Sm‐SeNPs(−) and Bm‐SeNPs(+) had similar effects on biofilm formation in the yeast strains, whereas the Ch‐SeNPs had no significant effect (Table 5). Previous investigation on the anti‐fungal effect of biogenic SeNPs synthesized by *Klebsiella pneumoniae* on *C. albicans* strain TUMS R152 showed a MIC of 2000 μg ml^−1^ (Kazempour *et al*., 2013), a value higher than the concentrations tested in this analysis. Contrarily to recent results evidencing a clear anti‐fungal activity of nanoparticles of different metals obtained by chemical synthesis (Lara *et al*., 2015), chemically synthesized SeNPs, although capable of antimicrobial activity against a number of nosocomial bacterial pathogens, were not efficacious towards the pathogenic yeasts tested, namely *C. albicans* and *C. parapsilosis*. In the present study, proteins associated to SeNPs of biogenic nature (Wang *et al*., 2010) apparently played a role in the anti‐fungal activity against yeasts that seemed — on the other hand — not to be affected by chemically synthesized SeNPs. The anti‐fungal activity of the biogenic SeNPs assayed was probably due to the disruptive interaction of their organic coat with the yeast outer wall layer, containing in *Candida* sp. mainly mannans and phosphorylated mannans. Permeabilization of the cell wall and subsequent disaggregation of the structural layers of the outer fungal cell wall are likely to be occurred. Nevertheless, the toxicity of metal nanoparticles towards yeasts and the associated toxic mechanisms remain controversial, and further investigation is needed in this area. Particularly, the cell wall constitutes the primary site for direct interaction with nanoparticles. Changes in yeast cell wall structure have been reported in response to many factors, e.g. growth conditions, mode of cultivation, compounds that block cell wall synthesis and anti‐fungal substances. However, there is a lack of information concerning changes in the cell wall composition in response to nanoparticle exposure (Sun *et al*., 2014).

**Table 5:** Percentage of biofilm synthesis inhibition in different fungal strains caused by Sm‐SeNPs, Bm‐SeNPs and Ch‐SeNPs

	15	30	50	60	100	120	250	325	400	500
Sm‐SeNPs (−) μg ml^−1^										
*Candida albicans*	40 ± 0.7	45 ± 0.5	61 ± 0.5	59 ± 0.7	60 ± 3	69 ± 1	60 ± 1	65 ± 2	77 ± 1	**94 ± 1**
*C. parapsilosis*	9 ± 3	42 ± 1	72 ± 1.5	60 ± 1.5	79 ± 0.5	70 ± 1	73 ± 1	72 ± 1	79 ± 3	**95 ± 1**
Bm‐SeNPs(−) μg ml^−1^										
*C. albicans*	51 ± 2	55 ± 2	60 ± 6.5	63 ± 3	69 ± 2	68 ± 2	74 ± 2.5	74 ± 1.5	**82 ± 0.5**	**93 ± 0.5**
*C. parapsilosis*	33 ± 2	49 ± 1	75 ± 1.5	70 ± 2.5	73 ± 0.5	70 ± 2	72 ± 3	71 ± 2	77 ± 1.5	**94 ± 0.5**
Ch‐SeNPs(−) μg ml^−1^										
*C. albicans*	0	0	0	0	0	0	0	0	5 ± 0.9	9 ± 0.7
*C. parapsilosis*	0	0	0	0	0	0	0	0	4 ± 1	5 ± 0.7

The percentage of inhibition was calculated relative to the quantity of biofilm formed by each strain in the absence of nanoparticles. Data are the average of results obtained in three different experiments. Percentages of inhibition higher than 80% are shown in bold.

### Degradation of *P. aeruginosa* and *Candida* spp. biofilms by SeNPs

We next investigated whether the SeNPs were able to cause the degradation of biofilms by measuring the amount of biofilm remaining after exposing the synthesized exopolysaccharide to different concentrations of biogenic and synthetic SeNPs (Table 6 and Table 7). The *P. aeruginosa* CFC20 biofilm was highly susceptible to SeNP‐induced disaggregation, resulting in 90% degradation in the presence of 50 μg ml^−1^ SeNPs, confirming that this strain is more susceptible to SeNPs than the other strains. The exposure of the other *P. aeruginosa* strains to 50–100 μg ml^−1^ SeNPs resulted in 50–70% biofilm degradation, and this did not increase at higher SeNP concentrations. Sm‐SeNPs(−) were slightly more efficient than Bm‐SeNPs(+) in the eradication of *P. aeruginosa* biofilms. Similarly, the biogenic SeNPs eradicated 45–60% of the yeast biofilms at the lowest SeNP doses (50–100 μg ml^−1^) and there was no improvement at higher doses. There was no significant difference in efficacy between the two types of biogenic SeNPs.

**Table 6:** Percentages of biofilm degradation in different bacteria strains caused by Sm‐SeNPs, Bm‐SeNPs and Ch‐SeNPs

Bacterial strain^a^	*Stenotrophomonas maltophilia*‐SeNPs (μg ml^−1^)				*Bacillus mycoides*‐SeNPs (μg ml^−1^)				Chemically synthesized SeNPs (μg ml^−1^)			
	50	100	250	500	50	100	250	500	50	100	250	500
*Pseudomonas aeruginosa* PAO1 (1.10)	73 ± 5	72 ± 2	76 ± 2	73 ± 5	52 ± 1	62 ± 4.5	57 ± 1	73 ± 6	16 ± 0.7	18 ± 4.2	37 ± 0.7	53 ± 1
*P. aeruginosa ATCC27853* (1.00)	49 ± 1.4	53 ± 1.4	53 ± 1.4	43 ± 0.7	31 ± 4.9	43 ± 1.4	51 ± 7	55 ± 7	23 ± 1.4	35 ± 1	40 ± 2.5	46 ± 0.7
*P. aeruginosa* INT (1.00)	63 ± 5.5	53 ± 0.5	61 ± 1.5	41 ± 4.5	65 ± 4.5	44 ± 0.5	58.2 ± 3	32 ± 1.5	15 ± 6.3	15 ± 6.3	8 ± 2.8	8 ± 4.5
*P. aeruginosa* CFC21 (1.20)	21 ± 1	45 ± 3.5	53 ± 5.5	63 ± 2	44 ± 4.5	16 ± 3	33 ± 1.5	61 ± 0.5	8 ± 1.4	5 ± 0.7	16 ± 1.4	1 ± 0.7
*P. aeruginosa* CFC20 (1.10)	**87 ± 3**	**84 ± 0.5**	**85 ± 1**	**85 ± 0.5**	**80 ± 1**	**86 ± 1**	**86 ± 1.7**	**82 ± 2**	17 ± 3.5	13 ± 2.8	29 ± 4.2	50 ± 2.8
*P. aeruginosa* CFCB (2.75)	53 ± 4	58 ± 3	56 ± 3	64 ± 2	25 ± 1	27 ± 5	51 ± 2.5	47 ± 3	0	4 ± 1.4	57 ± 1.4	72 ± 0.7
*P. aeruginosa* CFCA (3.00)	44 ± 2.5	44 ± 2.5	39 ± 1	53 ± 2	28 ± 4	20 ± 2.5	40 ± 2.5	53 ± 1	0	2 ± 0.7	66 ± 0.7	73 ± 0.7

a Quantity of biofilm in arbitrary unit.

The percentage of degradation was calculated relative to the quantity of biofilm formed by each strain in the absence of nanoparticles. Data represent the means of three different experiments. Percentages of biofilm degradation higher than 80% are shown in bold.

**Table 7:** Percentages of biofilm degradation in different fungal strains caused by Sm‐SeNPs, Bm‐SeNPs and Ch‐SeNPs

	15	30	50	60	100	120	250	325	400	500
Sm‐SeNPs(−) μg ml^−1^										
*Candida albicans*	0	5 ± 0.5	26 ± 2.5	30 ± 1	43 ± 2.5	30 ± 1.4	47 ± 3.5	49 ± 2	55 ± 3	60 ± 2
*C. parapsilosis*	0	0	52 ± 2	48 ± 1.4	48 ± 1.5	43 ± 1.5	48 ± 2.5	50 ± 0.5	59 ± 2.5	64 ± 2
Bm‐SeNPs(−) μg ml^−1^										
*C. albicans*	0	0	11 ± 2.5	12 ± 2	32 ± 2	41 ± 2	48 ± 1.5	51 ± 2	61 ± 1	60 ± 3.5
*C. parapsilosis*	0	0	48 ± 3	42 ± 1.5	38 ± 2	43 ± 1.5	47 ± 2	44 ± 1.5	47 ± 2	42 ± 2.5
Ch‐SeNPs(−) μg ml^−1^										
*C. albicans*	0	0	0	0	0	0	2	0	0	0
*C. parapsilosis*	0	0	0	0	1	0	0	0	0	0

The percentage of degradation was calculated relative to the quantity of biofilm formed by each strain in the absence of nanoparticles. Data represent the means of three different experiments.

The comparison of SeNPs from different origins provides insights into the optimization of strategies to inhibit the synthesis of biofilms or destroy biofilms that already exist. Biogenic SeNPs at concentrations of 50–100 μl ml^−1^ can suppress the synthesis of biofilms in three of the five clinical isolates of *P. aeruginosa* we tested, as well as two *Candida* species. SeNPs synthesized by the Gram‐negative bacterium *S. maltophilia* SeITE02 were slightly more efficacious than those produced by the Gram‐positive species *B. mycoides* SeITE01, although at concentrations ≥ 250 μg ml^−1^ both SeNPs were remarkably potent. Nevertheless, different clinical isolates of *P. aeruginosa* varied in susceptibility at physiologically compatible concentrations of biogenic SeNPs. The most potent disaggregation effects (45–70%) against both *P. aeruginosa* and yeast were observed at a SeNP concentration of 100 μg ml^−1^, and higher doses did not achieve greater activity. The disaggregation of *P. aeruginosa* biofilms by SeNPs also showed strain‐dependent efficacy, with strain CFC20 appearing particularly susceptible to biofilm disintegration probably due to the synthesis of a fragile exopolysaccharide matrix compared with the tougher and physically more resistant exopolysaccharide formations of the other isolates. Generally, the biogenic SeNPs at concentrations of 50–100 μg ml^−1^ (and the Sm‐SeNPs(−) in particular) were more effective in the destruction of existing biofilm structures than the inhibition of biofilm synthesis. This suggests that the anti‐biofilm mechanism of these nanomaterials is possibly targeted to a component of the mature biofilm structure. The biogenic SeNPs were more effective than synthetic counterparts, indicating that organic molecules on the surface are likely to contribute to their antimicrobial activities and enhance the effect of the inorganic selenium component.

### Effects of SeNPs on human cells

**Figure 3:**
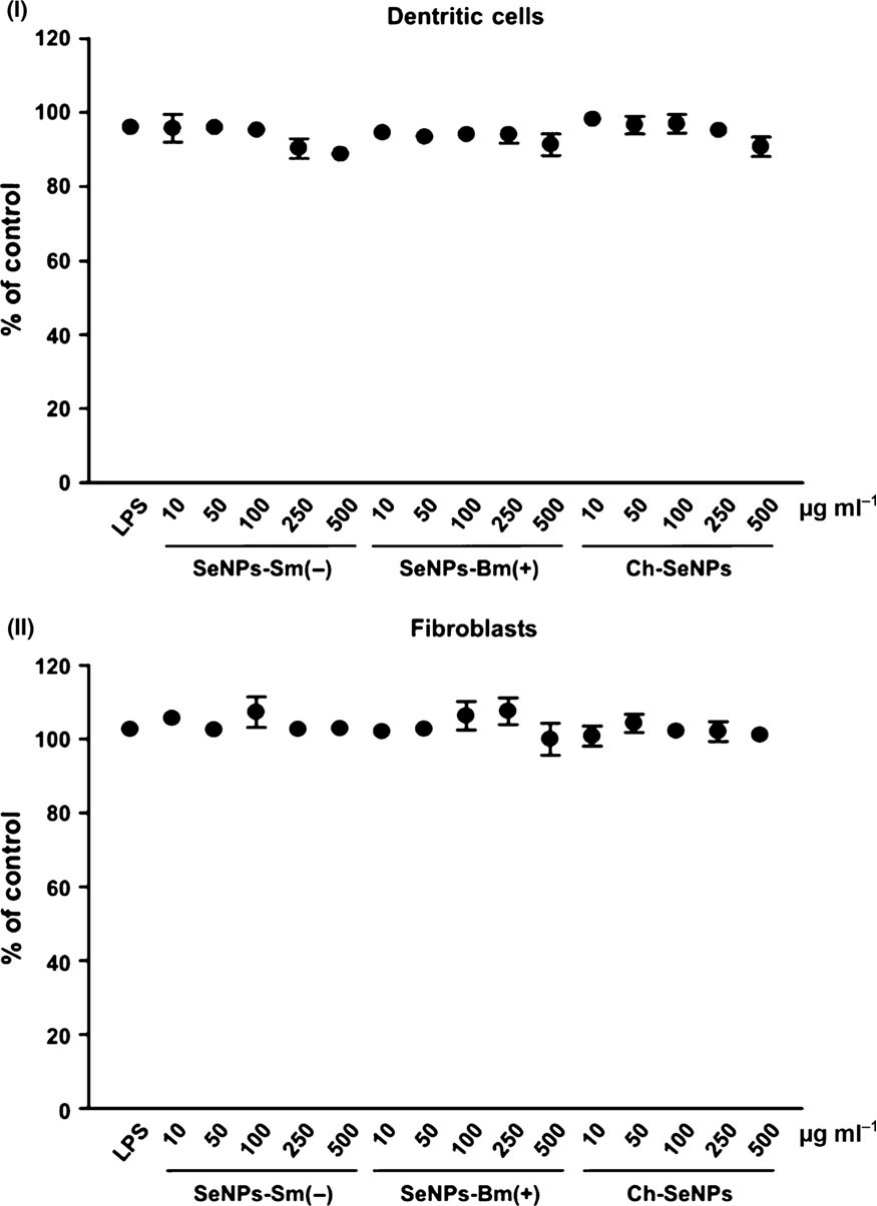
Evaluation of cell viability. DCs (I) and fibroblasts (II) were treated with the indicated concentrations of Sm‐SeNPs(−), Bm‐SeNPs(+) or Ch‐SeNPs for 24 h, followed by 4‐h incubation with Alamar blue. Cells were also incubated with 100 ng ml^−1^ LPS as a positive control. The values are expressed as the percentage of Alamar blue reduction relative to untreated cells (designated as 100%). Data are means ± SD of four experiments.

Some nanoparticles are known to be cytotoxic or to stimulate human cells, resulting in harmful off‐target effects (Chang and Gershwin, 2010; Di Gioacchino *et al*., 2011). The biogenic SeNPs in this study contain organic substances of bacterial origin, so it is necessary to determine whether they can damage human cells, or stimulate unanticipated effects, in the latter case particularly in immune system cells that recognize foreign structures and respond in order to neutralize and eliminate pathogens. We therefore investigated whether Sm‐SeNPs(−) and Bm‐SeNPs(+), as well as Ch‐SeNPs lacking biogenic molecules, affected the viability and activity of DCs. These are immune system cells that are fundamentally involved in the orchestration of inflammatory and immune response (Granucci *et al*., 2008; Schäkel, 2009). Human blood monocytes were cultured for 5 days in the presence of GM‐CSF and interleukin 4 (IL‐4) to obtain DCs, which were then challenged with different doses of SeNPs or with the bacterial immunostimulator LPS as a positive control. We also analysed the effect of SeNPs on the viability and activity of cultured human fibroblasts to determine whether SeNPs have adverse effects on non‐immune cells. Cell viability was assessed using Alamar blue, a colorimetric redox assay of metabolic activity. Different concentrations of the biogenic SeNPs (and Ch‐SeNPs) did not induce apoptosis in cultured DCs or fibroblasts, even at the highest dose of 500 μg ml^−1^, which is atypically high for standard *in vitro* cell stimulation protocols (Fig. 3).

We then investigated whether SeNPs stimulate DCs to release of pro‐inflammatory and immunostimulatory cytokines (particularly those involved in the activation of inflammatory and immune responses), such as IL‐12 which stimulates natural killer cells and T lymphocytes (Vignali and Kuchroo, 2012), IL‐8 which causes leucocyte chemotaxis and activation (Admyre *et al*., 2015), as well as IL‐6 and TNF‐α which elicit inflammation and the systemic acute phase reaction, characterized by fever, headache, anorexia, nausea, emesis and changes in the sleep–wake cycle (Elmquist *et al*., 1997; Suffredini *et al*., 1999).

**Figure 4:**
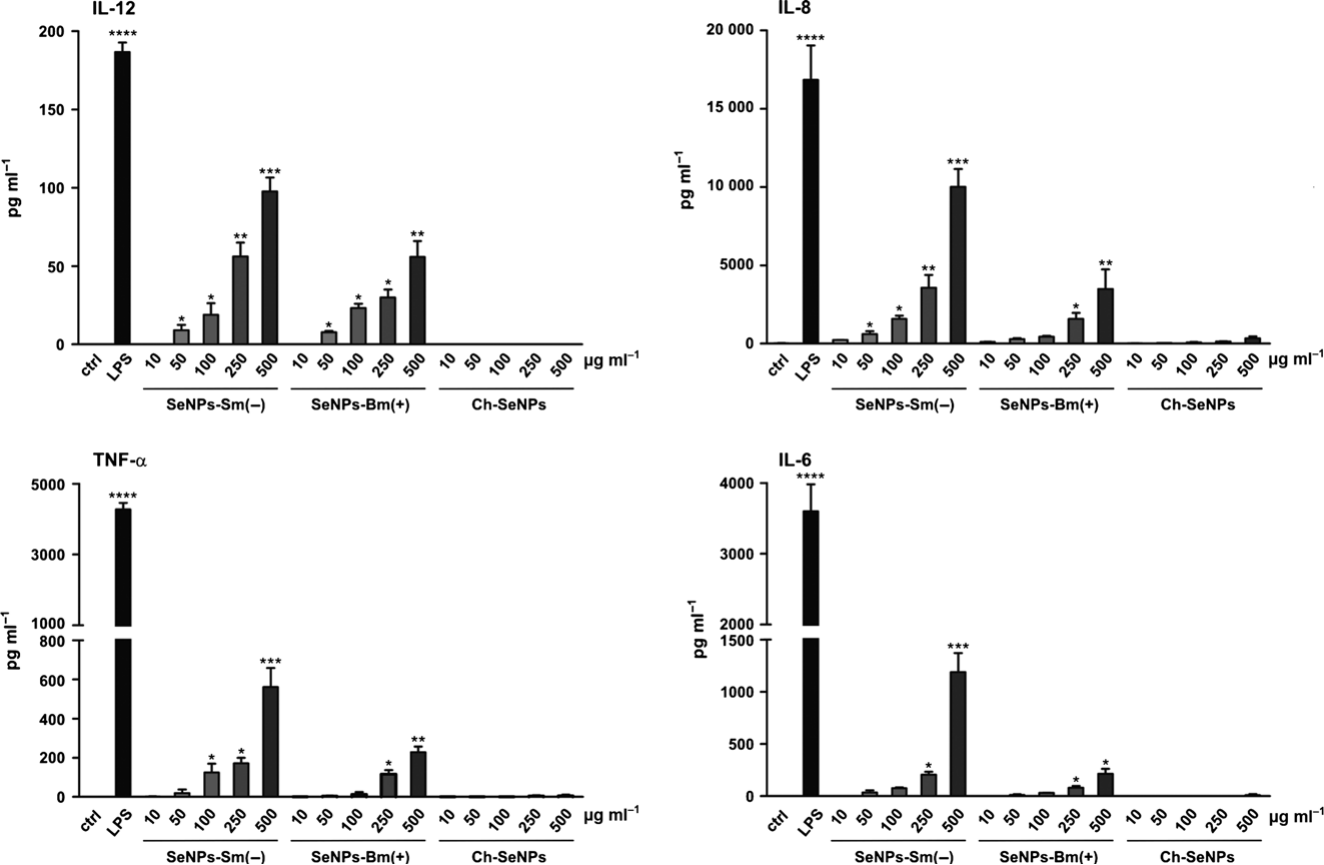
Quantification of cytokine production. DCs were challenged with the indicated amounts of Sm‐SeNPs(−), Bm‐SeNPs(+) or Ch‐SeNPs for 24 h. DCs were also activated with 100 ng ml^−1^ LPS as a positive control. The release of the indicated cytokines into the culture supernatants was evaluated by ELISA. The results are expressed as the mean value ± SD of three independent experiments. Statistical analysis: SeNP‐treated DCs versus untreated cells *P < 0.05, **P < 0.01, ***P < 0.001, ****P < 0.0001.

The analysis of DC culture supernatants by enzyme‐linked immunosorbent assay (ELISA) revealed that SeNPs did not induce a significant increase in the release of IL‐12, IL‐6, IL‐8 or tumor necrosis factor alpha (TNF‐α) until the doses reached 250–500 μg ml^−1^, which are unlikely to be achieved *in vivo* (Fig. 4). Cytokine release was stimulated more efficiently when DCs were challenged with Sm‐SeNPs(−) rather than Bm‐SeNPs(+), but bacterial LPS had a much more potent effect. Interestingly, the synthetic SeNPs did not induce the release of cytokines at any dose, suggesting that inorganic selenium is unable to stimulate human DCs alone and that organic molecules on the surface of biogenic SeNPs must be responsible for the observed effect. The identity of these organic molecules will be the subject of future research. We also explored whether SeNPs influence the release of pro‐inflammatory cytokines by human fibroblasts. We found that neither the biogenic SeNPs nor the Ch‐SeNPs induced the secretion of IL‐8, IL‐6 or TNF‐α by fibroblasts (data not shown), whereas 1 μg ml^−1^ LPS as a positive control induced human fibroblasts to secrete all three of these cytokines (data not shown).

Activated human DCs can also release remarkable quantities of reactive oxygen species that cause oxidative damage to neighbouring cells (Vulcano *et al*., 2004; Donini *et al*., 2007). We therefore investigated whether SeNPs were able to induce human DCs to produce oxygen radicals using the cytochrome *c* reaction assay to measure the quantity of superoxide anion release by the cells (Vulcano *et al*., 2004). We found that neither the biogenic SeNPs nor the Ch‐SeNPs induced the release of superoxide anions by DCs, whereas microbial cell wall glucan, which is known to promote the release of superoxide anions by immune system cells (Gantner *et al*., 2003; Donini *et al*., 2007) was able to achieve this effect (data not shown). Collectively, our results demonstrate that although SeNPs inhibit bacterial growth, they are unable to cause significant damage to human DCs and fibroblasts or to stimulate the release of cytokines or reactive oxygen species from the same cells, making them suitable candidates for further development as *in vivo* antimicrobial reagents.

## Conclusions

We have demonstrated that SeNPs synthesized by both *B. mycoides* SeITE01 and *S. maltophilia* SeITE02 possess potent antibacterial activities with low MIC values against a number of clinical strains of *P. aeruginosa,* but no biocidal effects against two clinical isolates of *C. albicans* and *C. parapsilosis*. To the best of our knowledge, this is the first report confirming that biogenic SeNPs are potentially suitable as antimicrobial agents against clinical strains isolated from patients with chronic lung diseases. We also show that the biogenic SeNPs can inhibit biofilm synthesis by *P. aeruginosa* and the two *Candida* species, and can efficiently disaggregate the mature exopolysaccharide matrix produced by these microorganisms. The antimicrobial potential of these biogenic SeNPs is greater than that of synthetic SeNPs, probably due to the presence of a bacterial protein layer coating the surface of the biogenic particles. Furthermore, SeNPs produced by the Gram‐negative species *S. maltophilia* are more potent antibacterial agents than those produced by the Gram‐positive species *B. mycoides*, suggesting that SeNPs with similar dimensions but originating from taxonomically distinct bacterial strains may show different activities, probably due to the different composition of their organic surface layer. Finally, neither the biogenic nor the synthetic SeNPs affected the viability of human DCs and fibroblasts, nor did they elicit the production of reactive oxygen species or the substantial secretion of pro‐inflammatory and immunostimulatory cytokines. Our data therefore suggest that the biogenic SeNPs are biocompatible structures that could be administered, either alone or in combination with antibiotics, in new therapeutic strategies to inhibit the growth of pathogens, including those resistant to antibiotics, or to facilitate the penetration of microbial biofilms.

## Experimental procedures

### Reagents

Analytical grade sodium selenite, selenous acid and reagents used for the chemical synthesis of SeNPs were purchased from Sigma‐Aldrich (Milan, Italy). RPMI 1640, Dulbecco's modified Eagle's medium (DMEM) and L‐glutamine were obtained from Lonza (Walkersville, MD, USA). Recombinant human GM‐CSF and human IL‐4 were purchased from Miltenyi Biotec GmbH (Bergisch Gladbach, Germany). Low‐endotoxin FBS and *Saccharomyces cerevisiae* glucan were obtained from Sigma‐Aldrich. All of the above reagents contained less than 0.125 endotoxin units per mL, as determined by the Limulus amebocyte lysate assay (Microbiological Associates, Walkersville, MD, USA). Ultrapure lipopolysaccharide (LPS 0111, B4 strain) from *Escherichia coli* was purchased from InvivoGen (San Diego, CA, USA). Mouse anti‐human CD1a (HI149) antibody was obtained from Becton–Dickinson (San Jose, CA, USA).

### Preparation of SeNPs

Two environmental isolates (Gram‐positive *B. mycoides* SeITE01 and the Gram‐negative *S. maltophilia* SeITE02) were used to produce the biogenic Bm‐SeNPs(+) and Sm‐SeNPs(−) respectively (Di Gregorio *et al*., 2005; Lampis *et al*., 2014). Sterile nutrient broth supplemented with 2 mM Na_2_SeO_3_ was inoculated to achieve final concentrations of 10^5^ and 10^7^ CFU ml^−1^ for *B. mycoides* SeITE01 and *S. maltophilia* SeITE02 respectively. The cultures were incubated aerobically at 27°C in a rotary shaker (150 r.p.m.) for 6 h (*B. mycoides* SeITE01) or 24 h (*S. maltophilia* SeITE02). Bacterial cells and nanoparticles were removed from the culture medium by centrifugation at 10 000 *g* for 10 min. The pellets were washed twice with 0.9% NaCl, suspended in Tris/HCl buffer (pH 8.2) and the cells were disrupted by ultrasonication at 100 W for 5 min. The suspension was then centrifuged at 10 000 *g* for 30 min to separate disrupted cells (pellet) from nanoparticles (supernatant). The nanoparticles were recovered after centrifugation at 40 000 *g* for 30 min, washed twice and suspended in deionized sterile water. Ch‐SeNPs were produced as described by Lin and Wang (2005), while Ch2‐SeNPs were produced as described by Li *et al*. (2010).

### Cell free extracts and CFX‐SeNPs preparation

*Stenotrophomonas maltophilia* SeITE02 and *B. mycoides* SeITE01 cells were grown for 24 h until stationary phase. They were then centrifuged at 3000 *g* for 15 min and washed twice with phosphate buffer saline. The pellet was resuspended in 100 mM Tris–HCl pH 7.4 and sonicated at 100 W five times for 5 min. Finally, unbroken cells were separated by centrifugation at 16 000 *g* for 30 min and the supernatant was recovered.

CFX‐SeNPs were then prepared by exposing Ch‐SeNPs to CFX of *S. maltophilia* SeITE02 or *B. mycoides* SeITE01 overnight in agitation. CFX‐SeNPs were recovered through centrifugation and washed twice with 100 mM Tris–HCl pH 7.4, as described by Dobias and coworkers (Dobias *et al*., 2011).

### Scanning electron microscopy

Both the biogenic and synthetic SeNPs were analysed by SEM. The nanoparticles were fixed, dehydrated through an increasing ethanol concentration series and dried in liquid CO_2_ using the critical point method. The particles were mounted on metallic specimen stubs and directly observed using an XL30 ESEM (FEI, Hillsboro, OR, USA) equipped with an EDAX micro‐analytical system, which was used to determine the elemental composition of the analysed nanoparticles.

### Dynamic light scattering

Dynamic light scattering analysis was carried out using a Zen 3600 Zetasizer Nano ZS (Malvern Instruments, Malvern, UK) equipped with a 633 nm helium–neon laser light source (4.0 mW), detecting scattering information at a fixed angle of 173°. Samples (300 μl) were transferred to a quartz cuvette (10 mm path length), and the mean size distribution and zeta potential were recorded at 25°C using the software provided by Malvern Instruments.

### Microbial strains and growth conditions

We used five *P. aeruginosa* clinical strains (INT, CFC20, CFC21, CFCA and CFCB) and two reference strains (PAO1 and PYO27853). *P. aeruginosa* INT is a multidrug‐resistant strain isolated from a urinary tract infection and it carries the class 1 integron containing multiple antibiotic‐resistant gene cassettes. Strains CFC20, CFC21, CFCB and CFCA were isolated from the sputum of patients from the Cystic Fibrosis Center of Verona, following the provision of written informed consent from the subjects enrolled in the study.

The bacterial strains were grown in tryptic soy broth (TSB) (Difco Laboratories, Detroit, MI, USA) or TSB medium supplemented with 1% glucose (TSB‐1% glucose).

Two yeast clinical strains, *Candida albicans* CVr‐21 and *Candida parapsilosis* CPVr‐5, isolated from vaginal swabs, were also included in the study and grown in Sabouraud medium. Cell growth was monitored with a LKB spectrophotometer at 640 nm (OD_640nm_).

### Nanoparticle susceptibility assay

The MIC of the different types of SeNPs tested (Ch‐SeNPs, Ch2‐SeNP, Sm‐SeNPs(−), Bm‐SeNPs(+), CFX(Sm)‐SeNPs, CFX(Bm)‐SeNPs) and cell free extracts (CFX(Sm) and CFX(Bm)) was first measured against the reference strain *P. aeruginosa* PAO1.

Afterwards, MICs were determined by the broth microdilution method (National Committee for Clinical Laboratory Standards, 2010) and were used to evaluate the susceptibility of the microbial strains. The susceptibility of the strains was confirmed in an agar diffusion assay by monitoring for the presence or absence of a bacterial growth inhibition halo surrounding wells containing different concentrations of SeNPs.

### Biofilm formation assay

Bacterial cells were grown at 37°C in TSB‐1% glucose and yeast cells were grown in Sabouraud medium until in each case they reached the exponential growth phase (OD_650nm_ = 0.4). Cells were then diluted in TSB‐1% glucose or Sabouraud medium to reach ~10^6^ CFU ml^−1^. We then inoculated sterile flat‐bottomed polystyrene CytoOne microtiter plates (Starlab, Milan, Italy) with 200 μl of each cell suspension or 200 μl of the medium without cells as a negative control. The anti‐biofilm properties of SeNPs were investigated by diluting them in growth medium to concentrations of 50, 100, 250 and 500 μg ml^−1^, and adding them to the microtiter wells. The plates were incubated aerobically at 37°C without agitation for 48 h to allow biofilm formation. After incubation, the planktonic cells and the growth medium were aseptically aspirated and the biofilm matrix washed with sterile saline solution and dried. Biofilm quantification was carried out by adding 100 μl of 1% methylene blue to each well and incubating for 15 min at room temperature. The wells were then slowly washed with sterile water and dried at 37°C. The methylene blue bound to the biofilm was extracted in 100 μl 70% ethanol and the absorbance was measured at 570 nm using an A3 Plate Reader (DAS, Rome, Italy). All experiments were performed in triplicate. Optical densities greater than 2, between 1 and 2 or between 0.5 and 1 optical units were considered to correspond to strong (S), medium (M) or low (L) biofilm production respectively.

### Biofilm disaggregation assay

Biofilm disaggregation triggered by SeNPs was measured by seeding the microbial suspensions into 96‐well microplates and incubating at 37°C to allow biofilm formation. After 48 h, the medium was aseptically aspirated. SeNPs were diluted in growth medium to concentrations of 50, 100, 250 and 500 μg ml^−1^, and were added to the wells. The prepared microplates were then incubated for 24 h at 37°C and the amount of biofilm was quantified as described above. All experiments were performed in triplicate.

### Preparation and culture of dendritic cells

After written informed consent was received from donors, and approval by the Ethical Committee (Prot. no. 5626, February 2nd 2012, and Prot. no. 43318, September 4th 2013), buffy coats from the venous blood of normal healthy volunteers were obtained from the Blood Transfusion Centre at the University Hospital of Verona. Peripheral blood mononuclear cells were isolated by Ficoll‐Hypaque and Percoll density gradient centrifugation (GE Healthcare Life Sciences, Little Chalfont, UK) and used for the immunomagnetic isolation (Miltenyi Biotec) of CD14^+^ cells as previously described (Zenaro *et al*., 2009). DCs were isolated by incubating 1 × 10^6^ monocytes per ml at 37°C in 5% CO_2_ for 5–6 days in six‐well tissue culture plates (Greiner Bio‐One, Nürtingen, Germany) in RPMI 1640 medium supplemented with heat‐inactivated 10% low‐endotoxin FBS, 2 mM L‐glutamine, 50 ng ml^−1^ GM‐CSF and 20 ng ml^−1^ IL‐4. The final DC population was 98% CD1a^+^, as measured by FACS analysis.

### Culture of fibroblasts

Human primary fibroblast CCD1112Sk cells (ATCC^®^ CRL‐2429) were purchased from ATCC (Manassas, VA, USA) and cultured in DMEM supplemented with 10% heat‐inactivated FBS plus 2 mM L‐glutamine at 37°C in 5% CO_2_.

### Quantification of cytokine production

Cytokine production in cell culture supernatants was determined by ELISA using Ready‐Set‐Go ELISA kits (eBioscience, San Diego, CA, USA) according to the manufacturer's instructions. We measured the levels of IL‐12 (range 4–500 pg ml^−1^), TNF‐α (range 4–500 pg ml^−1^) and IL‐6 (range 2–200 pg ml^−1^). The ELISA development kit (Mabtech, Nacka Strand, Sweden) was used to determine the level of IL‐8 (CXCL8 range 4–400 pg ml^−1^). Briefly, DCs were treated with different concentrations of SeNPs for 24 h, and then the supernatants were collected. DCs were also activated with 100 ng ml^−1^ LPS as a positive control. The plates were read at 450 nm with Victor^3^ 1420 Multilabel Counter (Perkin Elmer, Waltham, MA, USA).

### Cell viability evaluation

Cell viability was assessed using the AlamarBlue^®^ assay (Invitrogen, Thermo Fischer Scientific, Waltham, MA, USA) according to the manufacturer's instructions. After incubation for 24 h with SeNPs, the reagent was added to the culture medium a final concentration of 10% before measuring the absorbance at 570 and 600 nm.

### Superoxide anion production

The release of O_2_^−^ was estimated by cytochrome *c* reduction as previously described (Vulcano *et al*., 2004). Briefly, after cell culture, the medium was replaced with HBSS (pH 7.4) containing 80 μM ferricytochrome *c* (Sigma‐Aldrich) and the required stimulus. Cytochrome *c* reduction was determined by measuring absorbance at 550 nm using a Perkin‐Elmer Victor^3^ 1420 Multilabel Counter.

### Statistical analysis

Data are presented as means plus standard deviations. Statistical analysis, including two‐way analysis of variance, was carried out using GraphPad Prism v6.0 (GraphPad Software Inc., La Jolla, CA, USA).

## Conflict of interest

The authors have no conflict of interest to declare.

## References

[mbt212374-bib-0001] Admyre, C. , Axelsson, L.G. , von Stein, O. , and Zargari, A. (2015) Immunomodulatory oligonucleotides inhibit neutrophil migration by decreasing the surface expression of interleukin‐8 and leukotriene B4 receptors. Immunology 144: 206–217. 25100544 10.1111/imm.12368 PMC4298415

[mbt212374-bib-0002] Alexis, F. , Pridgen, E. , Molnar, L.K. , and Farokhzad, O.C. (2008) Factors affecting the clearance and biodistribution of polymeric nanoparticles. Mol Pharm 5: 505–515. 18672949 10.1021/mp800051m PMC2663893

[mbt212374-bib-0003] Beyth, N. , Houri‐Haddad, Y. , Domb, A. , Khan, W. , and Hazan, R. (2015) Alternative antimicrobial approach: nano‐antimicrobial materials. Evid Based Complement Alternat Med 246012: 1–16. 10.1155/2015/246012 PMC4378595 25861355

[mbt212374-bib-0004] Chang, C. , and Gershwin, M.E. (2010) Drugs and autoimmunity–a contemporary review and mechanistic approach. J Autoimmun 34: 266–275. 10.1016/j.jaut.2009.11.012 20015613

[mbt212374-bib-0005] Chudobova, D. , Cihalova, K. , Dostalova, S. , Ruttkay‐Nedecky, B. , Rodrigo, M.A. , Tmejova, F. , *et al* (2014) Comparison of the effects of silver phosphate and selenium nanoparticles on *Staphylococcus aureus* growth reveals potential for selenium particles to prevent infection. FEMS Microbiol Lett 351: 195–201. 24313683 10.1111/1574-6968.12353

[mbt212374-bib-0006] Ciofu, O. , Tolker‐Nielsen, T. , Jensen, P.Ø. , Wang, H. , and Høiby, N. (2015) Antimicrobial resistance, respiratory tract infections and role of biofilms in lung infections in cystic fibrosis patients. Adv Drug Deliv Rev 85: 7–23. 25477303 10.1016/j.addr.2014.11.017

[mbt212374-bib-0007] Di Gioacchino, M. , Tetrarca, C. , Lazzarin, F. , Di Giampaolo, L. , Sabbioni, E. , Boscolo, P. , *et al* (2011) Immunotoxicity of nanoparticles. Int J Immunopath Pharmacol 24: 65S–71S. 21329568

[mbt212374-bib-0008] Di Gregorio, S. , Lampis, S. , and Vallini, G. (2005) Selenite precipitation by a rhizospheric strain of *Stenotrophomonas* sp. isolated from the root system of *Astragalus bisulcatus*: a biotechnological perspective. Environ Int 31: 233–241. 15661289 10.1016/j.envint.2004.09.021

[mbt212374-bib-0009] Dobias, J. , Suvurova, E.I. , and Bernier‐Latmani, R. (2011) Role of proteins in controlling selenium nanoparticle size. Nanotechnology 22: 195605. 21430311 10.1088/0957-4484/22/19/195605

[mbt212374-bib-0010] Donini, M. , Zenaro, E. , Tamassia, N. , and Dusi, S. (2007) NADPH oxidase of human dendritic cells: role in *Candida albicans* killing and regulation by interferons, dectin‐1 and CD206. Eur J Immunol 37: 1194–1203. 17407098 10.1002/eji.200636532

[mbt212374-bib-0011] Elmquist, J.K. , Scammell, T.E. , and Saper, C.B. (1997) Mechanisms of CNS response to systemic immune challenge: the febrile response. Trends Neurosci 20: 565–570. 9416669 10.1016/s0166-2236(97)01138-7

[mbt212374-bib-0012] Gadakh, B. , and Van Aerschot, A. (2015) Renaissance in antibiotic discovery: some novel approaches for finding drugs to treat bad bugs. Curr Med Chem 22: 2140–2158. 25787965 10.2174/0929867322666150319115828

[mbt212374-bib-0013] Gantner, B.N. , Simmons, R.M. , Canavera, S.J. , Akira, S. , and Underhill, D.M. (2003) Collaborative induction of inflammatory responses by dectin‐1 and Toll‐like receptor 2. J Exp Med 197: 1107–1117. 12719479 10.1084/jem.20021787 PMC2193968

[mbt212374-bib-0014] Gill, E.E. , Franco, O.L. , and Hancock, R.E. (2015) Antibiotic adjuvants: diverse strategies for controlling drug‐resistant pathogens. Chem Biol Drug Des 85: 56–78. 25393203 10.1111/cbdd.12478 PMC4279029

[mbt212374-bib-0015] Grant, S.S. , and Hung, D.T. (2013) Persistent bacterial infections, antibiotic tolerance, and the oxidative stress response. Virulence 4: 273–283. 23563389 10.4161/viru.23987 PMC3710330

[mbt212374-bib-0016] Granucci, F. , Zanoni, I. , and Ricciardi‐Castagnoli, P. (2008) Central role of dendritic cells in the regulation and deregulation of immune responses. Cell Mol Life Sci 65: 1683–1697. 18327662 10.1007/s00018-008-8009-2 PMC11131678

[mbt212374-bib-0017] Habash, M.B. , Park, A.J. , Vis, E.C. , Harris, R.J. , and Khursigara, C.M. (2014) Synergy of silver nanoparticles and aztreonam against *Pseudomonas aeruginosa* PAO1 biofilms. Antimicrob Agents Chemother 58: 5818–5830. 25049240 10.1128/AAC.03170-14 PMC4187931

[mbt212374-bib-0018] Jorgensen, J.H. , and Ferraro, M.J. (2009) Antimicrobial susceptibility testing: a review of general principles and contemporary practices. Clin Infect Dis 49: 1749–1755. 19857164 10.1086/647952

[mbt212374-bib-0019] Kazempour, Z.B. , Yazdi, M.H. , Rafii, F. , and Shahverdi, A.R. (2013) Sub‐inhibitory concentration of biogenic selenium nanoparticles lacks post antifungal effect for *Aspergillus niger* and *Candida albicans* and stimulates the growth of *Aspergillus niger* . Iran J Microbiol 5: 81–85. 23466957 PMC3577562

[mbt212374-bib-0020] Kostakioti, M. , Hadjifrangiskou, M. , and Hultgren, S.J. (2013) Bacterial biofilms: development, dispersal, and therapeutic strategies in the dawn of the postantibiotic era. Cold Spring Harb Perspect Med 3: 1–23. 10.1101/cshperspect.a010306 PMC3683961 23545571

[mbt212374-bib-0021] Lampis, S. , Zonaro, E. , Bertolini, C. , Bernardi, P. , Butler, C.S. , and Vallini, G. (2014) Delayed formation of zero‐valent selenium nanoparticles by *Bacillus mycoides* SeITE01 as a consequence of selenite reduction under aerobic conditions. Microb Cell Fact 13: 1–14. 24606965 10.1186/1475-2859-13-35 PMC3975340

[mbt212374-bib-0022] Lampis, S. , Zonaro, E. , Bertolini, C. , Cecconi, D. , Monti, F. , Micaroni, M. , *et al* (2016) Selenite biotransformation and detoxification by *Stenotrophomonas maltophilia* SeITE02: novel clues on the route to bacterial biogenesis of selenium nanoparticles. J Hazard Mater in press. doi:10.1016/j.jhazmat.2016.02.035. 10.1016/j.jhazmat.2016.02.035 26952084

[mbt212374-bib-0023] Lara, H.H. , Romero‐Urbina, D.G. , Pierce, C. , Lopez‐Ribot, J.L. , Arellano‐Jiménez, M.J. , and Jose‐Yacaman, M. (2015) Effect of silver nanoparticles on *Candida albicans* biofilms: an ultrastructural study. J Nanobiotechnology 13: 1–12. 26666378 10.1186/s12951-015-0147-8 PMC4678641

[mbt212374-bib-0024] Li, Q. , Chen, T. , Yang, F. , Liu, J. , and Zheng, W. (2010) Facile and controllable one‐step fabrication of selenium nanoparticles assisted by L‐cysteine. Mater Lett 64: 614–617.

[mbt212374-bib-0025] Lin, Z.H. , and Wang, C.R.C. (2005) Evidence on the size‐dependent absorption spectral evolution of selenium nanoparticles. Mat Chem Phys 92: 591–594.

[mbt212374-bib-0026] Loza, K. , Diendorf, C. , Sengstock, C. , Ruiz‐Gonzalez, L. , Gonzalez‐Calbet, J.M. , Vallet‐Regi, M. , *et al* (2014) The dissolution and biological effects of silver nanoparticles in biological media. J Mater Chem B 2: 1634–1643. 10.1039/c3tb21569e 32261391

[mbt212374-bib-0028] Pelgrift, R.Y. , and Friedman, A.J. (2013) Nanotechnology as a therapeutic tool to combat microbial resistance. Adv Drug Deliv Rev 65: 1803–1815. 23892192 10.1016/j.addr.2013.07.011

[mbt212374-bib-0029] Penesyan, A. , Gillings, M. , and Paulsen, T.T. (2015) Antibiotic discovery: combating bacterial resistance in cells and in biofilm communities. Molecules 20: 5286–5298. 25812150 10.3390/molecules20045286 PMC6272253

[mbt212374-bib-0030] Schäkel, K. (2009) Dendritic cells–why can they help and hurt us. Exp Dermatol 18: 264–273. 19183400 10.1111/j.1600-0625.2008.00823.x

[mbt212374-bib-0031] Shakibaie, M. , Forootanfar, H. , Golkari, Y. , Mohammadi‐Khorsand, T. , and Shakibaie, M.R. (2015) Anti‐biofilm activity of biogenic selenium nanoparticles and selenium dioxide against clinical isolates of *Staphylococcus aureus, Pseudomonas aeruginosa,* and *Proteus mirabilis* . J Trace Elem Med Biol 29: 235–241. 25175509 10.1016/j.jtemb.2014.07.020

[mbt212374-bib-0032] Suffredini, A.F. , Fantuzzi, G. , Badolato, R. , Oppenheim, J.J. , and O'Grady, N.P. (1999) New insights into the biology of the acute phase response. J Clin Immunol 19: 203–214. 10471974 10.1023/a:1020563913045

[mbt212374-bib-0033] Sun, M. , Yu, Q. , Hu, M. , Hao, Z. , Zhang, C. , and Li, M. (2014) Lead sulfide nanoparticles increase cell wall chitin content and induce apoptosis in *Saccharomyces cerevisiae* . J Haz Mat 273: 7–16. 10.1016/j.jhazmat.2014.03.008 24704549

[mbt212374-bib-0034] Tomita, R.J. , de Matos, R.A. , and Vallim, M.A. (2014) A simple and effective method to synthesize fluorescent nanoparticles using tryptophan and light and their lethal effect against bacteria. J Photochem Photobiol, B 140: 157–162. 25129701 10.1016/j.jphotobiol.2014.07.015

[mbt212374-bib-0035] Tran, P.A. , and Webster, T.J. (2011) Selenium nanoparticles inhibit *Staphylococcus aureus* growth. Int J Nanomedicine 6: 1553–1558. 21845045 10.2147/IJN.S21729 PMC3152473

[mbt212374-bib-0036] Vignali, D.A. , and Kuchroo, V.R. (2012) IL‐12 family cytokines: immunological playmakers. Nat Immunol 13: 722–728. 22814351 10.1038/ni.2366 PMC4158817

[mbt212374-bib-0037] Vulcano, M. , Dusi, S. , Lissandrini, D. , Badolato, R. , Mazzi, P. , Riboldi, E. , *et al* (2004) Toll receptor‐mediated regulation of NADPH oxidase in human dendritic cells. J Immunol 173: 5749–5756. 15494527 10.4049/jimmunol.173.9.5749

[mbt212374-bib-0038] Wang, T. , Yang, L. , Zhang, B. , and Liu, J. (2010) Extracellular biosynthesis and transformation of selenium nanoparticles and application in H_2_O_2_ biosensor. Colloids Surf B Biointerfaces 80: 94–102. 20566271 10.1016/j.colsurfb.2010.05.041

[mbt212374-bib-0039] Xiangqian, L. , Huizhong, X. , Zhe‐Sheng, C. , and Guofang, C. (2011) Biosynthesis of nanoparticles by microorganisms and their applications. J Nanomater 270974: 1–16. doi:10.1155/2014/359316.

[mbt212374-bib-0040] Zenaro, E. , Donini, M. , and Dusi, S. (2009) Induction of Th1/Th17 immune response by *Mycobacterium tuberculosis*: role of dectin‐1, mannose receptor, and DC‐SIGN. J Leukocyte Biol 86: 1393–1401. 19773555 10.1189/jlb.0409242

[mbt212374-bib-0041] Zonaro, E. , Lampis, S. , Turner, R.J. , Qazi, S.J. , and Vallini, G. (2015) Biogenic selenium and tellurium nanoparticles synthesized by environmental microbial isolates efficaciously inhibit bacterial planktonic cultures and biofilm. Front Microbiol 6: 1–11. 26136728 10.3389/fmicb.2015.00584 PMC4468835

